# Impact of postoperative complications after primary tumor resection on survival in patients with incurable stage IV colorectal cancer: A multicenter retrospective cohort study

**DOI:** 10.1002/ags3.12433

**Published:** 2021-01-25

**Authors:** Yusuke Fujita, Koya Hida, Nobuaki Hoshino, Yoshiharu Sakai, Tsuyoshi Konishi, Akiyoshi Kanazawa, Michitoshi Goto, Shuji Saito, Tadashi Suda, Masahiko Watanabe, Japan Society of Laparoscopic Colorectal Surgery

**Affiliations:** ^1^ Department of Surgery Kyoto University Graduate School of Medicine Kyoto Japan; ^2^ Department of Surgery Osaka Red Cross Hospital Osaka Japan; ^3^ Department of Surgical Oncology The University of Texas MD Anderson Cancer Center Houston TX USA; ^4^ Department of Surgery Shimane Prefectural Central Hospital Shimane Japan; ^5^ Department of Coloproctological Surgery Juntendo University Faculty of Medicine Tokyo Japan; ^6^ Division of Surgery Gastrointestinal Center Yokohama Shin‐Midori General Hospital Yokohama Japan; ^7^ Suda Medical Clinic Kawasaki Japan; ^8^ Department of Surgery Kitasato University School of Medicine Kanagawa Japan

**Keywords:** colorectal neoplasms, complication, primary tumor resection, stage IV, survival

## Abstract

**Aims:**

Primary tumor resection for patients with incurable stage IV colorectal cancer can prevent tumor‐related complications but may cause postoperative complications. Postoperative complications delay the administration of chemotherapy and can lead to the spread of malignancy. However, the impact of postoperative complications after primary tumor resection on survival in patients with incurable stage IV colorectal cancer remains unclear. Therefore, this study aimed to investigate how postoperative complications after primary tumor resection affect survival in this patient group.

**Methods:**

We reviewed data on 966 patients with stage IV colorectal cancer who underwent palliative primary tumor resection between January 2006 and December 2007. We examined the association between major complications (National Cancer Institute Common Terminology Criteria for Adverse Events v3.0 grade 3 or more) and overall survival using Cox proportional hazard model and explored risk factors associated with major complications using multivariable logistic regression analysis.

**Results:**

Ninety‐three patients (9.6%) had major complications. The 2‐year overall survival rate was 32.7% in the group with major complications and 50.3% in the group with no major complications. Patients with major complications had a significantly poorer prognosis than those without major complications (hazard ratio: 1.62; 95% confidence interval: 1.21‐2.18; *P* < .01). Male, rectal tumor, and open surgery were identified to be risk factors for major complications.

**Conclusions:**

Postoperative complications after primary tumor resection was associated with decreased long‐term survival in patients with incurable stage IV colorectal cancer.

## Graphical abstract

This study aimed to investigate the impact of postoperative complications after primary tumor resection on survival in patients with incurable stage IV colorectal cancer. We reviewed data on 966 patients with stage IV colorectal cancer who underwent palliative primary tumor resection. Our results suggest that major postoperative complications after primary tumor resection worsened the prognosis of patients with incurable stage IV colorectal cancer.

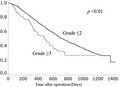

## 1 INTRODUCTION

Colorectal cancer is the fourth most commonly diagnosed cancer and the second leading cause of cancer deaths worldwide.^1^ Approximately 20% of all patients with colorectal cancer are diagnosed with stage IV cancer, and approximately 80% of those patients cannot undergo curative resection of the distant metastasis.^2^, ^3^

The effectiveness of palliative primary tumor resection (PTR) for incurable stage IV patients is still controversial.^4^, ^5^, ^6^ PTR could prevent tumor‐related complications, such as intestinal obstruction, perforation, bleeding, or fistula.^7^, ^8^, ^9^ Conversely, PTR may cause postoperative complications that requires time for the patients to recover from, which subsequently delays the administration of systemic chemotherapy and these delays can lead to the systemic spread of malignancy.^10^, ^11^, ^12^

Postoperative complications can worsen the long‐term survival as well as short‐term outcomes. Previous studies reported that postoperative complications decrease survival after curative surgery; however, only a few reports have focused on PTR for patients with incurable stage IV colorectal cancer.^13^, ^14^, ^15^

This study aimed to investigate the impact of postoperative complications after PTR on survival in patients with incurable stage IV colorectal cancer.

## 2 METHODS

### 2.1 Study design and setting

This is a retrospective cohort study using data from the Japan Society of Laparoscopic Colorectal Surgery (JSLCS). The JSLCS retrospectively collected data on patients with stage IV colorectal cancer who underwent primary tumor resection at 41 participating hospitals between January 2006 and December 2007. This database did not include patients who underwent resection of metastasis with curative intent, irrespective of whether this was simultaneous or two‐stage. All surgeons were experienced in laparotomy, and most have performed over 100 laparoscopic surgeries. Consecutive patient demographics and clinicopathological data, including patient characteristics, surgical findings, perioperative treatment, tumor stage, and survival, were collected retrospectively. The protocol for this research project has been approved by a suitably constituted Ethics Committee of Kyoto University (E631) and it conforms to the provisions of the Declaration of Helsinki.

### 2.2 Eligibility

Patients with stage IV disease were included in this study. From them, we excluded patients with postoperative 30‐day or in‐hospital death.

### 2.3 Postoperative complications

Postoperative complications were graded according to the National Cancer Institute Common Terminology Criteria for Adverse Events v3.0 (CTCAE).^16^ Major complications were defined as CTCAE grade 3 or 4. No major complications were defined as no complications or CTCAE grade 1 or 2. Surgical site infections and remote site infections were defined as infectious complications and any other complications were defined as non‐infectious complications.^17^

### 2.4 Statistical analysis

Continuous variables were compared using the Mann‐Whitney *U* test. Categorical valuables were compared using the Fisher's exact test.

Overall survival (OS) was defined as the time between the date of primary tumor resection and the date of death. Survival curves were created by Kaplan‐Meier estimates, and they were compared by the log‐rank test. Multivariable cox regression models were used to examine the association between major complications and OS, adjusting for all variables. Subgroup analyses were performed to explore the differences in impact of major complications on OS according to age, American Society of Anesthesiologists ‐ Physical Status (ASA‐PS), and the number of organs with residual tumor.

Multivariable logistic regression models were performed to explore factors associated with major complications. Of all the variables assessed, other than postoperative treatments, we selected variables that had a *P* value of <.1 in the univariable analysis or clinically relevant factors such as age, ASA‐PS, emergency operation, tumor depth, and lymph node metastasis.

All *P* values were two‐sided, and *P* values <.05 were considered statistically significant. All statistical analyses were performed using JMP Statistical Software Version 14 (SAS‐Institute Inc, Cary, NC, USA).

## 3 RESULTS

### 3.1 Patient characteristics

Of 972 patients with stage IV disease, 966 patients were included after excluding those with postoperative death. Patient characteristics are shown in Table 1. Among them, 93 patients (9.6%) had major complications. The proportion of males, rectal tumor, open surgery, operative time ≥240 minutes, and bleeding of ≥100 mL tended to be higher in the major complications group.

**TABLE 1:** Patient characteristics

Factor	n	Complication^a^			
		Grade ≤ 2		Grade ≥ 3	
Category		n	(%)	n	(%)
Total	966	873	(90.4)	93	(9.6)
Clinical findings					
Age (years)					
<70	639	582	(91.1)	57	(8.9)
≥70	327	291	(89.0)	36	(11.0)
Sex					
Male	548	482	(88.0)	66	(12.0)
Female	417	390	(93.5)	27	(6.5)
Body mass index (kg/m^2^)					
<25	783	708	(90.4)	75	(9.6)
≥25	171	156	(91.2)	15	(8.8)
ASA‐PS					
I‐II	868	786	(90.6)	82	(9.4)
III‐IV	88	77	(87.5)	11	(12.5)
Emergency operation					
−	907	823	(90.7)	84	(9.3)
+	59	50	(84.7)	9	(15.3)
Previous laparotomy					
−	713	647	(90.7)	66	(9.3)
+	233	212	(91.0)	21	(9.0)
Preoperative chemotherapy					
−	908	820	(90.3)	88	(9.7)
+	58	53	(91.4)	5	(8.6)
Preoperative radiotherapy					
−	956	864	(90.4)	92	(9.6)
+	10	9	(90.0)	1	(10.0)
CEA (ng/mL)					
<5	189	178	(94.2)	11	(5.8)
≥5	763	684	(89.6)	79	(10.4)
Tumor location					
Right colon	323	299	(92.6)	24	(7.4)
Left colon	452	412	(91.2)	40	(8.8)
Rectum	177	148	(83.6)	29	(16.4)
Perioperative intestinal stenosis					
−	474	429	(90.5)	45	(9.5)
+	462	420	(90.9)	42	(9.1)
Symptom(s) relating to primary tumor^b^					
Asymptomatic	432	393	(91.0)	39	(9.0)
Symptomatic	505	456	(90.3)	49	(9.7)
Surgical findings					
Surgical approach					
Open	737	654	(88.7)	83	(11.3)
Lap	229	219	(95.6)	10	(4.4)
Additional operation					
−	770	700	(90.9)	70	(9.1)
+	196	173	(88.3)	23	(11.7)
Operative time (min)					
<240	696	638	(91.7)	58	(8.3)
≥240	267	232	(86.9)	35	(13.1)
Bleeding (mL)					
<100	387	362	(93.5)	25	(6.5)
≥100	570	502	(88.1)	68	(11.9)
Intraoperative complications					
−	956	863	(90.3)	93	(9.7)
+	10	10	(100.0)	0	(0.0)
Tumor development					
Tumor depth					
≤T3	525	479	(91.2)	46	(8.8)
T4	441	394	(89.3)	47	(10.7)
Lymph node metastasis					
−	157	146	(93.0)	11	(7.0)
+	787	706	(89.7)	81	(10.3)
Hepatic metastasis					
−	269	245	(91.1)	24	(8.9)
+	691	622	(90.0)	69	(10.0)
Peritoneal metastasis					
−	683	622	(91.1)	61	(8.9)
+	277	246	(88.8)	31	(11.2)
Other distant metastasis					
−	548	492	(89.8)	56	(10.2)
+	409	373	(91.2)	36	(8.8)
Number of organs with residual tumors					
1	625	570	(91.2)	55	(8.8)
≥2	341	303	(88.9)	38	(11.1)
Postoperative treatment					
Postoperative chemotherapy					
−	174	146	(83.9)	28	(16.1)
+	792	727	(91.8)	65	(8.2)
Days to start chemotherapy^c^		31	(20‐45)	44	(33‐60)
Other postoperative therapies					
−	840	753	(89.6)	87	(10.4)
+	126	120	(95.2)	6	(4.8)

Abbreviations: ASA‐PS, American Society of Anesthesiologists ‐ Physical Status; CEA, carcinoembryonic antigen.

^a^ Postoperative complications were graded according to National Cancer Institute Common Terminology Criteria for Adverse Events v3.0.

^b^ Symptom(s) relating to primary tumor are defined as symptoms indicating anemia (hemoglobin, <9 mg/L) and intestinal stricture, signifying that the colon fiberscope could not be inserted on the oral side due to colorectal tumor.

^c^ Median (interquartile range).

Introduction of postoperative chemotherapy was delayed in patients with major complications compared to those with no major complications (44 vs 31 days, *P* < .01). Regarding the duration of the delay by each major complication, anastomotic leak was 48 days (interquartile range [IQR], 41‐86), ileus/obstruction was 42 days (IQR, 27‐63), wound infection was 36 days (IQR 25‐61), lung infection was 36 days (IQR, 30‐48), and intra‐abdominal infection was 35 days (IQR 18‐53).

### 3.2 Details of postoperative complications

The characteristics of postoperative complications are described in Table 2. There was a total of 246 complications, including 92 grade 3 complications and 11 grade 4 complications. Anastomotic leak, ileus/obstruction, and wound infection were the most common complications. Infectious major complications were found in 53 patients and non‐infectious major complications were found in 46 patients. Multiple complications were found in 21 patients.

**TABLE 2:** Postoperative complications

CTCAE grade	Grade 1‐2	Grade 3	Grade 4	Total	(%)
Anastomotic leak	13	21	2	36	(3.7)
Ileus/Obstruction	36	32	2	70	(7.2)
Wound infection	59	9	1	69	(7.1)
Intra‐abdominal infection	11	6	1	18	(1.9)
Urinary dysfunction	6	3	0	9	(0.9)
Bleeding	1	2	0	3	(0.3)
Stroke	0	0	3	3	(0.3)
PE/DVT	0	2	0	2	(0.2)
Lung infection	1	6	1	8	(0.8)
Others	16	11	1	28	(2.9)
Total	143	92	11	246	
Multiple complications: 21 cases					

Abbreviations: CTCAE, National Cancer Institute Common Terminology Criteria for Adverse Events v3.0; DVT, deep venous thrombosis; PE, pulmonary embolism.

### 3.3 Factors associated with major complications

In the logistic regression model, male, rectal tumor, and open surgery were significantly associated with major complications (Table 3).

**TABLE 3:** Factors associated with major complications

Variables	Category			Adjusted OR	95% CI	*P* value
Clinical findings						
Age (years)	≥70	/	<70	1.36	[0.86‐2.16]	.19
Sex	Male	/	Female	1.76	[1.09‐2.85]	.02
ASA‐PS	III‐IV	/	I‐II	1.16	[0.55‐2.45]	.70
Emergency operation	+	/	−	1.53	[0.65‐3.57]	.33
Tumor location	Rectum	/	Colon	1.92	[1.14‐3.24]	.01
Surgical findings						
Surgical approach	Open	/	Lap	2.55	[1.24‐5.23]	.01
Operative time (min)	≥240	/	<240	1.53	[0.91‐2.56]	.11
Bleeding (mL)	≥100	/	<100	1.17	[0.68‐2.01]	.57
Tumor development						
Tumor depth	T4	/	≤T3	1.17	[0.75‐1.84]	.48
Lymph node metastasis	+	/	−	1.44	[0.73‐2.83]	.29

Abbreviations: ASA‐PS, American Society of Anesthesiologists ‐ Physical Status; CI, confidence interval; OR, odds ratio.

### 3.4 Survival analysis

**FIGURE 1:**
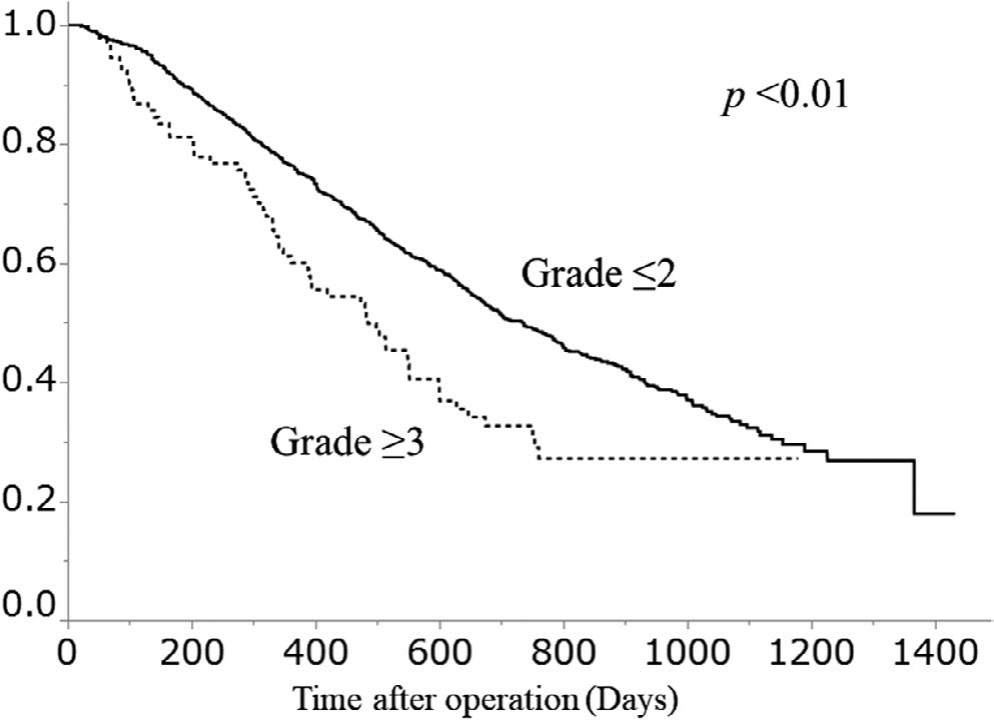
Overall survival of patients stratified by the presence or absence of major postoperative complications

**FIGURE 2:**
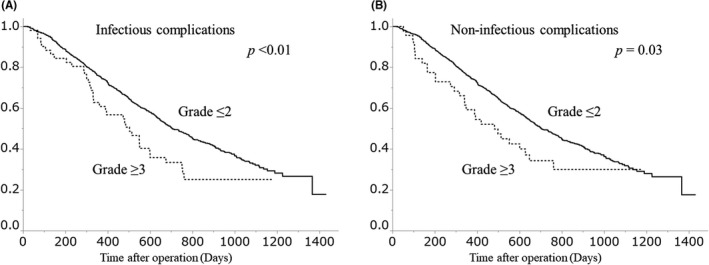
Overall survival of patients based on type of complications: (A) Infectious complications; (B) Non‐infectious complications

**FIGURE 3:**
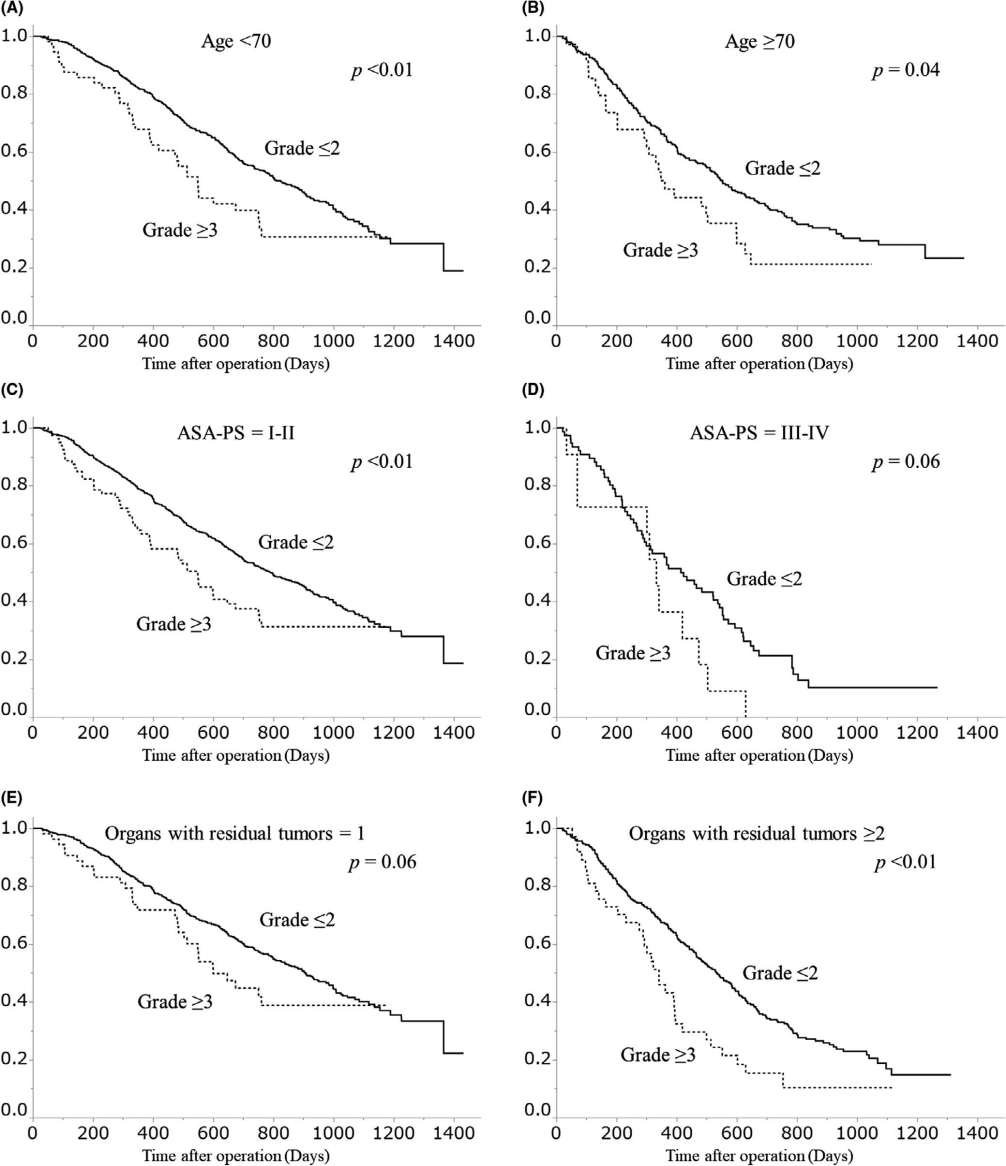
Subgroup analyses on overall survival stratified by the presence or absence of major postoperative complications, according to age, American Society of Anesthesiologists ‐ Physical Status (ASA‐PS), and the number of organs with residual tumor: (A) Age < 70, (B) Age ≥ 70, (C) ASA‐PS = I‐II, (D) ASA‐PS = III‐IV, (E) Organs with residual tumors = 1, (F) Organs with residual tumors ≥ 2

The OS curves of patients stratified by major or no major postoperative complications are shown in Figure 1. The 2‐year OS rate was 50.3% in the group with no major complications and 32.7% in the group with major complications. OS was significantly lower in the group of major complications (*P* < .01). The OS curves of patients based on type of complication (infectious or non‐infectious) are shown in Figure 2. Subgroup analyses according to age, ASA‐PS, and the number of organs with residual tumor are shown in Figure 3.

In the Cox proportional hazards model, major complications were significantly associated with poor OS (hazard ratio: 1.62; 95% confidence interval: 1.21‐2.18; *P* < .01), after adjusting for all factors of patient characteristics (Table 4).

**TABLE 4:** Association between major complications with overall survival

Variables	Category			Adjusted HR	95% CI	*P* value
Postoperative complications^a^	Grade ≥ 3	/	Grade ≤ 2	1.62	[1.21‐2.18]	<.01
Clinical findings						
Age (years)	≥70	/	<70	1.37	[1.12‐1.67]	<.01
Sex	Male	/	Female	1.11	[0.92‐1.34]	.27
Body mass index (kg/m^2^)	≥25	/	<25	0.87	[0.68‐1.11]	.28
ASA‐PS	III‐IV	/	I‐II	1.50	[1.10‐2.01]	.01
Emergency operation	+	/	−	0.81	[0.53‐1.19]	.29
Previous laparotomy	+	/	−	1.22	[0.99‐1.51]	.06
Preoperative chemotherapy	+	/	−	1.29	[0.84‐1.90]	.24
Preoperative radiotherapy	+	/	−	1.05	[0.38‐2.48]	.91
CEA (ng/mL)	≥5	/	<5	1.33	[1.03‐1.73]	.03
Tumor location Left colon	Left colon	/	Right colon	0.79	[0.64‐0.98]	.04
Tumor location Rectum	Rectum	/	Right colon	0.99	[0.73‐1.32]	.93
Preoperative intestinal stenosis	+	/	−	1.22	[1.01‐1.48]	.03
Surgical findings						
Surgical approach	Open	/	Lap	0.97	[0.77‐1.24]	.83
Additional operation	+	/	−	0.87	[0.68‐1.10]	.24
Operative time (min)	≥240	/	<240	0.67	[0.52‐0.86]	<.01
Bleeding (mL)	≥100	/	<100	0.95	[0.77‐1.17]	.62
Intraoperative complications	+	/	−	1.60	[0.73‐3.51]	.24
Tumor development						
Tumor depth	T4	/	≤T3	1.21	[1.00‐1.46]	.05
Lymph node metastasis	+	/	−	1.66	[1.25‐2.20]	<.01
Hepatic metastasis	+	/	−	1.89	[1.49‐2.39]	<.01
Peritoneal metastasis	+	/	−	1.28	[1.02‐1.60]	.03
Other distant metastasis	+	/	−	1.42	[1.18‐1.72]	<.01
Postoperative treatment						
Postoperative chemotherapy	+	/	−	0.49	[0.39‐0.63]	<.01
Other postoperative therapy	+	/	−	0.40	[0.29‐0.56]	<.01

Abbreviations: ASA‐PS, American Society of Anesthesiologists ‐ Physical Status; CEA, carcinoembryonic antigen; CI, confidence interval; HR, hazard ratio.

^a^ Postoperative complications were graded according to National Cancer Institute Common Terminology Criteria for Adverse Events v3.0.

## 4 DISCUSSION

In this study, we showed that patients with stage IV colorectal cancer with major postoperative complications after PTR had a poor prognosis. In addition, we identified that male sex, rectal tumor, and open surgery were significantly associated with major complications.

Several mechanisms have been proposed to explain the negative impact of postoperative complications on oncologic outcomes. Postoperative complications lead to the omission or delay in administering postoperative chemotherapy.^15^, ^18^, ^19^ In this study, patients in the major complications group had an increased delay for the start of postoperative chemotherapy compared to patients with no major complications. In addition, postoperative complications cause inhibition of the adaptive immune response secondary to tissue damage, anesthesia, blood transfusion, and, in particular, infectious complications.^13^, ^20^ The negative effect of complications was reportedly greater in more advanced stages.^21^, ^22^ Several studies have reported on the impact of postoperative complications on long‐term survival after colorectal cancer surgery, including curative surgery for patients with stages I‐III diseases (hazard ratio: 1.24‐1.36)^13^, ^21^ and curative hepatic resection for colorectal liver metastasis (hazard ratio: 1.41‐1.52).^23^, ^24^ Our results for incurable colorectal cancer (hazard ratio: 1.62) corresponded to the previous studies.

This study attempted to explore the difference in the impact of postoperative complications on prognosis based on the type of complication. Previous studies have demonstrated that the infectiousness of complications worsened patients' prognoses.^13^, ^23^ On the other hand, the results of this study are consistent with those of a systematic review by McSorley et al,^17^ which reported that the prognostic impact of infectious complications and that of non‐infectious complications were similar. In addition, this study investigated the impact of major complications on OS stratified by age, ASA‐PS, and the number of organs with residual tumors. The prognostic impact of major complications appeared similar in all the subgroups.

Many studies have reported on the risk factors for postoperative complications in patients undergoing curative surgery such as age, sex, ASA‐PS, emergency operation, tumor location, surgical approach, and disease stage.^13^, ^19^, ^25^ On the other hand, few studies have referred to incurable patients. Stillwell et al reported sex, advanced local disease, repeat operations, elevated urea levels, and emergency operations as risk factors for the complications in incurable surgery.^26^ However, in that study, the colon and rectum were not distinguished, and laparoscopic surgery was not performed. In this study, male, rectal tumor, and open surgery were factors associated with major complications. PTR is sometimes inevitable for patients with incurable colorectal cancer because of tumor‐related symptoms, such as intestinal stenosis and bleeding.^27^ Surgeons should consider such risk factors when performing PTR. Laparoscopic surgery would be a good option, as it reportedly has a lower complication rate and similar prognosis compared to open surgery in previous studies on curative surgery.^25^, ^28^, ^29^, ^30^

The strength of this study is that it is the first study to clarify the association between postoperative complications and survival following PTR for patients with incurable colorectal cancer. Additionally, we utilized a large cohort of patients with incurable stage IV colorectal cancer to adjust for as many confounders as possible. However, this study has some limitations. This is a retrospective cohort study, and thus there might be unmeasurable confounding factors that could influence the study results. In addition, we could not obtain information on patient comorbidities and details of postoperative therapies. Nevertheless, we believe that the results of our study provide important information for surgeons engaging the treatment of incurable colorectal cancer.

In conclusion, the major postoperative complications after PTR worsened the prognosis of patients with incurable stage IV colorectal cancer.

## DISCLOSURE

Funding: This study was supported by the Mitsubishi Foundation (30327).

Conflict of Interest: The authors declare that they have no conflict of interest.
